# What IJCP authors think about open access: exploring one possible future for publishing clinical research in a general and internal medicine journal

**DOI:** 10.1111/j.1742-1241.2011.02884.x

**Published:** 2012-02

**Authors:** C Graf

**Affiliations:** Publisher, International Journal of Clinical Practice, Editorial Director, Wiley-Blackwell, John Wiley & Sons155 Cremorne Street, Richmond, Victoria 3121, Australia Email: cgraf@wiley.com Twitter: @ijcpeditors

This editorial shares the questions that IJCP is, at the time of this publication, asking research authors about ‘open access’. It aims to prompt discussion about open access, and explains some of the challenges that switching to open access presents to a general and internal medicine journal like IJCP. It invites you to share your insights, using the approaches described below. It explains how you will be able to read the results of our research.

## An important trend

Open access publishing has changed, perhaps forever, the way readers access articles in some high-quality, peer-reviewed journals.

**Figure 1:**
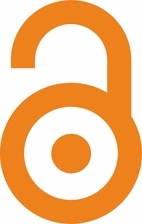
Open access logo as an example of these authors re-using work published by the original authors using one of the Creative Commons licenses (1)

The benefits to authors are derived from removing the ‘pay to read’ barriers that traditionally sit between published articles and the readers who want to read them. Arguably this gives greater exposure for authors’ work. Replacing traditional copyright attribution with Creative Commons-based licensing – another component of publishing open access – means that authors’ work is arguably more straightforward to re-use [for example, Figure 1 (1)]. Also, although this is controversial, there may be a possible citation advantage (2,3).

The cost to authors, though, is real. Whereas there are frequently no publication costs for authors who publish in traditional subscription journals, publishing in open access journals commonly involves a mandatory publication fee. Sometimes, this fee is paid by research funding bodies (some of which make unrestricted access a condition of their funding), such as Wellcome Trust, US National Institutes of Health, UK Medical Research Council and some corporations, including pharmaceutical companies. Other times authors pay from their own pockets.

The cost to publishers is also real. The open access model means that publishers can no longer collect the subscription revenues that traditionally sustain their journals and may mean that they instead switch to reliance on authors paying.

Equally critical, publishers who make the switch from a subscription to an open access approach run the serious risk of alienating their authors, who may not believe that the benefits of open access outweigh the costs, and who instead take their articles to different journals, which continue with the traditional approach.

## Hybrid: nice but (unless something changes) suboptimal

Some journals, like IJCP, continue to offer authors a hybrid option: open access for individual articles within a journal where all other articles are available via subscription. This approach was developed to explore the possible transition between models, from subscription to open access. While showing some signs of success, the hybrid approach is (and may remain) suboptimal for some research funders, and the spectre of ‘double dipping’ raises its head (4).

## Full open access: what IJCP authors think

At the time of this publication, and for another 6 months, we will ask all authors who submit papers to IJCP to answer two questions about open access (Table 1). The answers to these questions will tell us whether or not these authors believe the possible benefits of open access publishing might outweigh the costs.

**Table 1:** The questions that we are, at time of publication, asking all IJCP authors to answer

Question	Statement	Format for answer
Do you agree or disagree with this statement	‘I would still choose to submit my work to IJCP if IJCP in the future required all authors to pay an open access fee for publication and then made all its content open access and free to read online’	Yes/No
	We welcome your general comments (e.g. you may want to comment on what you think a suitable open access fee might be, considering our benchmark of $3000)	Free text
Do you agree or disagree with this statement	‘I would not publish my work in any open access journal (including IJCP if IJCP required me to pay an open access fee) because the costs to me or my research funder would outweigh any benefits’	Yes/No
	We welcome your general comments	Free text

Then, using these answers, the IJCP team will consider whether or not IJCP should become an open access journal. If we decide to take this course, IJCP would start requiring payment of open access fees from authors for every article we agree to publish, although we would have a fee-waiver option for certain cases. We would no longer collect subscription revenues for the articles we publish online and these articles would become free to read by anyone. We would calculate our open access publication fee in such a way as to replace our current subscription revenue.

## Discussion: letters, Twitter

We wrote this editorial to explain our approach, and to extend discussion to a wider audience beyond those authors who happen to submit to IJCP in the next 6 months. We encourage you to share your thoughts and join the debate, by submitting to IJCP a ‘Letter to the Editor’ (approximately 200 words, at our usual submission website) explaining what you think about open access, and/or the traditional subscription model, and why. We will review all letters, as is normal, and publish in the journal those that add insight. We also encourage those readers who are Twitterers to discuss open access with us on Twitter:@ijcpeditors. And we will also be happy to receive communications by email.

## Results: to be published

By answering our questions or by joining in the conversation, you will be taking part in an interesting and highly topical debate (in the publishing world, at least), as well as playing an active role in helping decide how IJCP approaches open access in the future. We will aggregate and analyse the responses that we receive from authors (Table 1), and will share those results with you in a future editorial. Like this editorial, we will publish the results in an article that is free to read online. To receive this news as soon as it is published, please register with IJCP online to get new content alerts (5).

## Disclosures

CG is employed by John Wiley & Sons, and as such benefits from the success of the company’s publishing programme. CG publishes clinical and research journals including a number for Societies and Royal Colleges in Australia and New Zealand, the International Journal of Clinical Practice, the global Wiley open access journals in health sciences, is the treasurer of the Committee on Publication Ethics (a UK registered charity), and leads the Wiley-Blackwell publication ethics programme.
